# *In vitro* antimicrobial activity of crude propolis extracts and fractions

**DOI:** 10.1093/femsmc/xtad010

**Published:** 2023-04-20

**Authors:** Alhassan Sa-eed, Eric S Donkor, Reuben E Arhin, Patience B Tetteh-Quarcoo, Simon K Attah, Daniel E K Kabotso, Fleischer C N Kotey, Nicholas T K D Dayie

**Affiliations:** Department of Medical Microbiology, University of Ghana Medical School, P. O. Box KB 4236, Korle Bu, Accra, Ghana; Department of Medical Laboratory Technology, Accra Technical University, P. O. Box GP 561, Barnes Road, Accra, Ghana; Department of Medical Microbiology, University of Ghana Medical School, P. O. Box KB 4236, Korle Bu, Accra, Ghana; Department of Medical Microbiology, University of Ghana Medical School, P. O. Box KB 4236, Korle Bu, Accra, Ghana; Department of Science Laboratory Technology, Accra Technical University, P. O. Box GP 561, Barnes Road, Accra, Ghana; Department of Medical Microbiology, University of Ghana Medical School, P. O. Box KB 4236, Korle Bu, Accra, Ghana; Department of Medical Microbiology, University of Ghana Medical School, P. O. Box KB 4236, Korle Bu, Accra, Ghana; Baldwin University College, P. O. Box 19872, Osu, Accra, Ghana; Department of Basic Sciences, School of Basic and Biomedical Sciences, University of Health and Allied Sciences, PMB 31, Ho, Ghana; Department of Medical Microbiology, University of Ghana Medical School, P. O. Box KB 4236, Korle Bu, Accra, Ghana; FleRhoLife Research Consult, P.O. Box TS 853, Teshie, Accra, Ghana; Department of Medical Microbiology, University of Ghana Medical School, P. O. Box KB 4236, Korle Bu, Accra, Ghana

**Keywords:** antimicrobial, activity, crude, propolis, extracts, fractions

## Abstract

The search for antimicrobials in propolis presents a new dimension for addressing the problem of antimicrobial drug resistance. The aim of this study was to determine the antimicrobial activity of extracts of crude propolis collected from different regions in Ghana and their active fractions. The antimicrobial activity of the extracts, as well as that of the chloroform, ethyl acetate, and petroleum ether fractions of the active samples were determined using the agar well diffusion method. The minimum inhibitory concentration (MIC) and the minimum bactericidal concentration (MBC) of the most active fractions were determined. The various crude propolis extracts frequently produced zones of inhibition against *Staphylococcus aureus* (17/20) than *Pseudomonas aeruginosa* (16/20), and *Escherichia coli* (1/20) test isolates. Chloroform and ethyl acetate solvents produced fractions possessing greater antimicrobial activity than the petroleum ether fraction. The mean MIC range of the most active fractions was greatest for *S. aureus* (76.0 ± 34.8–48.0 ± 33.0 mg/ml) than for *P. aeruginosa* (40.8 ± 33.3–30.4 ± 6.7 mg/ml) and *E. coli*, as was the mean MBC. Propolis has antimicrobial potential, and hence should be exploited as an alternative for the treatment of bacterial infections.

## Teaser

Propolis can be exploited as an alternative drug for the treatment of infections caused by bacteria.

## Introduction

Medicinal plants and their derivatives play a vital role in covering the basic health needs in under-resourced settings (WHO 1998). One of such plant derivatives is bee propolis (bee glue), a resin-type substance made up of a complex mixture of several substances mainly collected from tree sap and leaf buds by the honeybee, *Apis mellifera* (Orsi et al. 2005). It has been considered a good candidate for the prevention and treatment of infectious diseases (Orsi et al. 2005). The increasing use of propolis in modern traditional medicine has attracted researchers to investigate its chemical composition and antimicrobial properties (Amoros et al. 1992, Huang et al. 2014). This is because propolis is of plant-based origins and may offer the possibility of discovering unique and important phytochemicals for the treatment of infectious diseases.

Currently, antimicrobial drug resistance is having a serious impact on healthcare and economies around the globe (WHO 2014). In Ghana, antimicrobial drug therapy constitutes a major form of treatment for infectious diseases (Opintan et al. 2015). The Antimicrobial Drug use, Monitoring, and Evaluation of Resistance project (ADMER 2015) revealed the seriousness of the increasing resistance of bacteria to conventional antimicrobial drugs in Ghana. Across the southern, central, and northern sectors of Ghana, varied levels of resistance have been recorded. Recently, a multicenter antimicrobial resistance surveillance study in the country revealed alarming rates of resistance against Gram-negative bacteria isolated from blood stream infections (Donkor et al. 2023). Succinctly put, the enormity of the antimicrobial resistance menace has limited the therapeutic use of antimicrobial drugs for the treatment and control of many infectious diseases (Opintan et al. 2015, Addae-Nuku et al. 2022, Baah et al. 2022, Dayie et al. 2022, Dwomoh et al. 2022, Kotey et al. 2022, Donkor et al. 2023). This phenomenon threatens the success of medical interventions, and presents a set of specific challenges for clinical, therapeutic, and public health measures both nationally and internationally (WHO 2014). As a result, there has been a continuous search for antimicrobial compounds present in natural products. Concentrated whole plant extracts have been adopted in forms such as infusions, creams, and pills as part of a holistic treatment plan to address the antimicrobial drug resistance menace. Propolis has gained attention as part of the search for alternative antimicrobial agents from natural products to combat drug-resistant bacteria (Ali et al. 2018).

The search for antimicrobials in propolis presents a new dimension for addressing the problem of resistance to antimicrobial drugs. The aim of this study was to determine the antimicrobial activity of crude propolis extracts and selected solvent-derived fractions collected from different regions in Ghana.

## Materials and methods

### Sampling sites

Propolis samples were collected from selected commercial beekeepers from each of the then 10 regions of Ghana covering a total land area of 238 535 km^2^. Beekeepers in Ghana [Upper West Region (UWR), Upper East Region (UER), Northern Region (NR), Brong Ahafo Region (BAR), Ashanti Region (AR), Eastern Region (ER), Western Region (WR), Greater Accra Region (GAR), Volta Region (VR), and Central Region (CR)] are known for capturing wild bees and do not use hive chemicals (Llorens-Picher et al. 2017); hence selection was based on the vegetation cover and regional location of the hive. One hive was selected from each region.

### Preparation of the crude extract solutions

The crude extract solutions were prepared using the method described by Fabricant and Fansworth (2001). The collected propolis were sorted to remove pieces of wood, embalmed insects, and other animals. The samples were air dried under a shed and pulverized. For each sample, 30 g was weighed and dissolved in absolute ethanol. This was kept for 72 hours and filtered twice using Whatman No.1 filter paper. Each filtrate was evaporated using a rotary evaporator to obtain a solid mass, which was subsequently dried in a desiccator. Using 5% dimethyl sulfoxide (DMSO) (Daejung, Korea) as a solvent, solutions of 64 mg/ml were prepared for each dry solid mass obtained.

### Antimicrobial screening of the crude extract solutions

The prepared solutions were tested for antimicrobial activity using the agar well diffusion method. Clinical isolates characterized by multidrug resistance, such as *Staphylococcus aureus* (*S. aureus*), *Escherichia coli* (*E. coli*), and *Pseudomonas aeruginosa* (*P. aeruginosa*) obtained from the Microbiology Laboratory of the Center for Plant Medicine Research, Mampong, were used to prepare inocula of cell densities 10^6^ cells/ml using the direct colony suspension method. These were used alongside *S. aureus* ATCC 25923, *E. coli* NCTC 13351, and *P. aeruginosa* ATCC 27853 quality control strains.

For each prepared inoculum, a sterile swab stick was moistened in the inoculum and used to inoculate the surface of Mueller-Hinton agar plates. A 6-mm diameter sterile cock borer was used to create wells of 3 mm depth in each Mueller-Hinton agar plate and filled with 100 μl of each crude extract solution; 5% DMSO was included for testing as a negative control. The plates were incubated at 37 °C for 18 hours, and the diameter of each zone of inhibition was measured (Asiedu-Gyekye et al. 2005, Dayie et al. 2008, CLSI 2016).

### Fractionation of the selected crude extract dried masses

Based on observation of the antimicrobial activities from the screening, the NR, ER, VR, and AR crude extract dried masses were selected. Using a separatory funnel, analytical grades of chloroform (CH), ethyl acetate (EA), and petroleum ether (PE) (British Drug House and Fruka) solvents were used for the extraction of polar, medium polar, and nonpolar bioactive components, respectively. A rotary evaporator was used to remove the extraction solvents from each fraction to obtain dry soluble masses of the extracts.

### Antimicrobial activity of the fractions

The dry masses obtained were resuspended in 5% DMSO. The antimicrobial activity of each fraction was determined in biological duplicates using the agar well diffusion method, as previously described for the screening. Each well of the Mueller-Hinton agar plate was filled with 100 μl of the fraction and the plates were incubated at 37 °C for 18 hours. The diameter of each zone of inhibition was measured (Dayie et al. 2008, CLSI 2016). Kirby-Bauer disk diffusion tests using vancomycin, amikacin, and clindamycin commercial reference antibiotic disks were utilized as positive controls.

Fractions showing a zone of inhibition >15 mm against at least two test bacteria were selected on observation, and their minimum inhibitory concentrations (MIC) were determined using the macrobroth dilution method (CLSI 2016).

**Figure 1.**
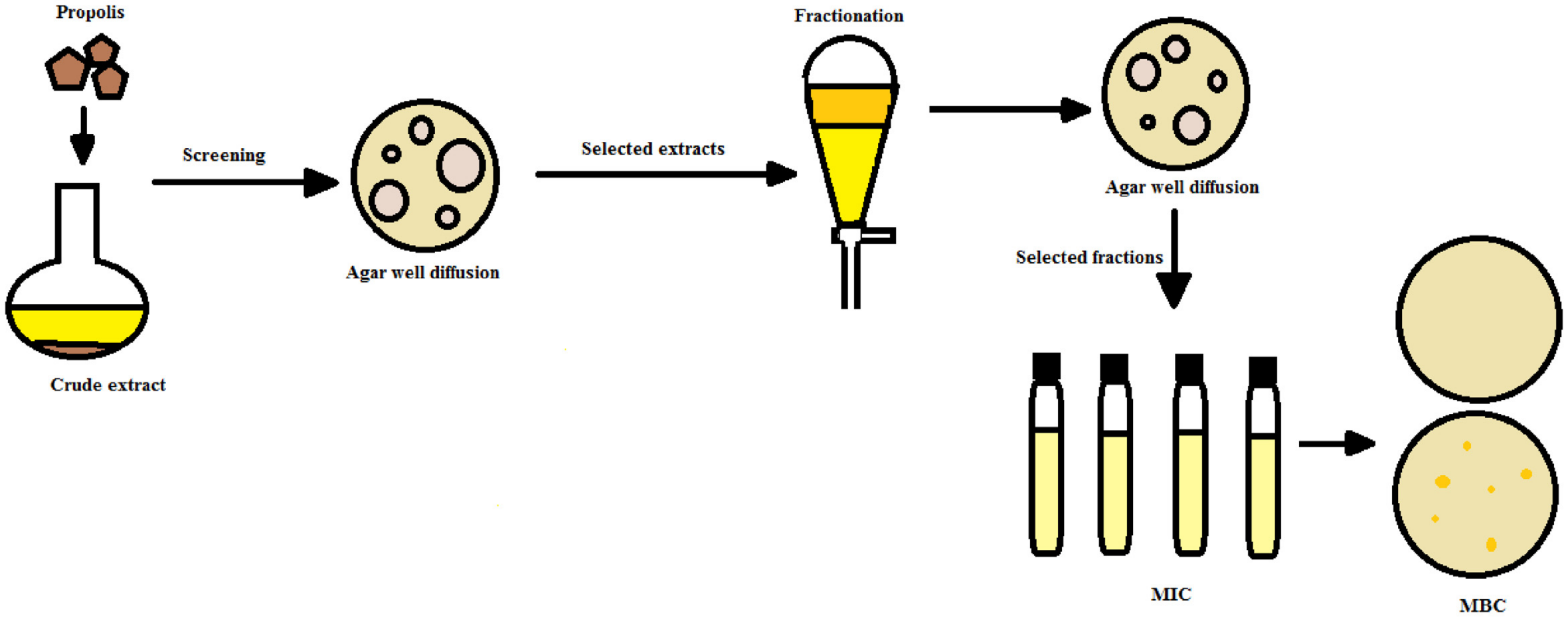
Illustration of the study methodology.

The lowest concentration of the fractions that prevented visible growth of the test bacteria in the Mueller-Hinton broths was subcultured onto Mueller-Hinton agar plates. The plates were incubated at 37 °C for 18 hours and checked for growth. The lowest concentration of each fraction for which there was no growth after subculturing on the Mueller-Hinton agar plates was recorded as the minimum bactericidal concentration (MBC) for the fraction. An illustration of the study methodology is presented in Fig. 1.

### Data analysis

StataMP14 was used to analyze the data. Means were computed for zones of inhibition produced by the crude propolis, propolis fractions, and reference antibiotic disks. Unpaired *t*-tests were performed to determine the significance of the difference between the means of the crude extract zones for each clinical isolate and their corresponding type strain. One sample *t*-test was performed to determine the significance of the difference between the means of the crude propolis extract zones and the reference zones. One-way ANOVA was used to determine if there was any statistically significant difference across the MICs and MBCs of the effective crude propolis fractions. *Post hoc* analysis was performed by the Bonferroni test. *P* values <.05 were considered as significant evidence against the null hypothesis.

## Results

### Screening of the crude propolis extract

**Figure 2.**
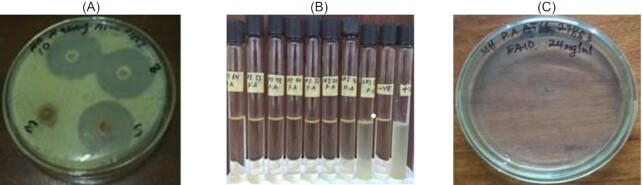
(A) Zones of inhibition produced by the propolis extract, (B) MIC of the propolis fraction for *P. aeruginosa*, and (C) MBC of the propolis fraction for *P. aeruginosa*.

The crude propolis extracts produced zones of inhibition in 17 of the 20 tests (85%) involving clinical and type strain isolates of *S. aureus* (Table 1; Fig. 2A). The greatest antimicrobial activity against clinical *S. aureus* strains was produced by the NR (23.5 ± 0.5 mm) and AR (23.5 ± 0.5 mm) crude propolis extracts. The GAR, BAR, and ER crude propolis extracts did not produce any antimicrobial activity against the clinical *S. aureus* strain. The greatest antimicrobial activity of the crude propolis extracts against the type strain *S. aureus* was produced by the AR (25.0 ± 1.0 mm) and NR (24.0 ± 1.0 mm) crude extracts. There was no significant difference (*p* = .1541) between the mean antimicrobial activity of the crude propolis extract for the clinical *S. aureus* (11.9 ± 9.5 mm) and the type strain *S. aureus* (15.6 ± 5.8 mm).

**Table 1.** Zones of inhibition of the crude propolis extracts.

Propolis	Zone diameter (mm)					
Source	*S. aureus*		*P. aeruginosa*		*E. coli*	
	Clinical	Type strain	Clinical	Type strain	Clinical	Type strain
UWR	11.0 ± 0.0	11.0 ± 0.0	11.0 ± 0.0	19.5 ± 0.5	0.0 ± 0.0	0.0 ± 0.0
UER	10.0 ± 0.0	10.0 ± 1.0	10.0 ± 0.0	17.5 ± 0.5	0.0 ± 0.0	0.0 ± 0.0
NR	23.5 ± 0.5	24.0 ± 1.0	12.0 ± 0.0	27.5 ± 0.5	0.0 ± 0.0	0.0 ± 0.0
BAR	0.0 ± 0.0	12.5 ± 0.5	0.0 ± 0.0	19.0 ± 1.0	0.0 ± 0.0	0.0 ± 0.0
AR	23.5 ± 0.5	25.0 ± 1.0	12.5 ± 0.0	30.5 ± 1.5	0.0 ± 0.0	0.0 ± 0.0
ER	0.0 ± 0.0	11.5 ± 0.5	0.0 ± 0.0	29.5 ± 0.5	0.0 ± 0.0	0.0 ± 0.0
WR	11.5 ± 0.5	11.0 ± 0.0	0.0 ± 0.0	21.5 ± 1.5	0.0 ± 0.0	0.0 ± 0.0
CR	19.5 ± 1.5	19.5 ± 0.5	10.0 ± 1.0	19.5 ± 1.5	0.0 ± 0.0	0.0 ± 0.0
GAR	0.0 ± 0.0	11.5 ± 0.5	0.0 ± 0.0	17.5 ± 0.5	0.0 ± 0.0	0.0 ± 0.0
VR	20.0 ± 0.0	19.5 ± 0.5	13.0 ± 0.0	24.0 ± 1.0	0.0 ± 0.0	19.5 ± 0.5
Mean ± SD	11.9 ± 9.5	15.6 ± 5.8	6.9 ± 5.7	22.0 ± 4.4	0.0 ± 0.0	2.0 ± 6.2
5% DMSO	0.0 ± 0.0	0.0 ± 0.0	0.0 ± 0.0	0.0 ± 0.0	0.0 ± 0.0	0.0 ± 0.0

Key: UWR—Upper West Region, UER—Upper East Region, NR—Northern Region, BAR—Brong Ahafo Region, ER—Eastern Region, GAR—Greater Accra Region, WR—Western Region, AR—Ashanti Region, CR—Central Region, and VR—Volta Region.

The crude propolis extracts produced zones of inhibition in 16 of the 20 tests (80%) involving clinical and type strain *P. aeruginosa* (Table 1). The least antimicrobial activity against type strain *P. aeruginosa* was produced by the UWR (11.0 ± 0.0 mm) and UER (10.0 ± 0.0 mm) crude propolis extracts. The greatest antimicrobial activity of the crude propolis extracts against the clinical *P. aeruginosa* isolate was produced by the VR (13.0 ± 0.0 mm) and AR (12.5 ± 0.5 mm) crude extracts. The BAR, ER, and WR crude extracts did not produce any antimicrobial activity against the clinical *P. aeruginosa* isolate. The greatest antimicrobial activity against the type strain *P. aeruginosa* was produced by the AR (30.5 ± 1.5 mm) and NR (27.5 ± 0.5 mm) crude propolis extracts. The least antimicrobial activity of the crude extract against the type strain *P. aeruginosa* was produced by the GAR (18 mm) and UER (18 mm) crude extracts. The mean zone of inhibition of the crude propolis extract for the clinical *P. aeruginosa* (6.9 ± 5.7 mm) was significantly lesser (*p* = .001) than that of the type strain *P. aeruginosa* (22.0 ± 4.4 mm).

The crude propolis extracts produced zones of inhibition in 1 of the 20 tests (5%) involving the clinical and type strain *E. coli* (Table 1). They did not show any antimicrobial activity against the clinical *E. coli* (0.0 ± 0.0 mm) and the type strain *E. coli*, except in the case of the VR (19.5 ± 0.5 mm) crude extract. There was no significant difference (*p* = .1717) between the mean antimicrobial activity of the crude propolis extract for the clinical *E. coli* (0.0 ± 0.0 mm) and the type strain *E. coli* (2.0 ± 6.2 mm).

### Zones of inhibition for the crude propolis extracts and the reference antibiotic disks

For the clinical *S. aureus* isolate, the mean zone of inhibition of the crude extract (11.9 ± 9.5 mm) was not significantly different (*p* = .4807) from the amikacin reference zone (12 mm) (Table 2). It was also not significantly different (*p* = .7231) from the clindamycin reference zone (10 mm).

**Table 2.** Mean zones of inhibition for the crude extracts and reference zones with interpretive categories against the tested bacteria.

Test bacteria	Zone of inhibition (mm)					*P* value of means			
	Extract zone	Reference zone (Interpretive Category)				Reference disk			
	Mean ± SD	Amk	Van	Cli	Mean ± SD	Amk	Van	Cli	Mean ± SD
*S. aureus* (Clinical)	11.9 ± 9.5	12 (R)	18 (NA)	10 (S)	20.0 ± 9.2	.4807	.0356	.7231	.0120
*S. aureus* (Type strain)	15.6 ± 5.8	21 (S)	16 (NA)	19 (I)	18.7 ± 2.5	.0081	.4064	.0471	.0625
*P. aeruginosa* (Clinical)	6.9 ± 5.7	21 (S)	15 (NA)	20 (NA)	18.7 ± 3.2	.0000	.0010	.0000	.0001
*P. aeruginosa* (Type strain)	22.6 ± 4.4	20 (S)	18 (NA)	28 (NA)	22.0 ± 5.3	.8606	.9855	.0006	.3899
*E. coli* (Clinical)	0.0 ± 0.0	20 (S)	10 (NA)	16 (NA)	15.3 ± 9.0	NA	NA	NA	NA
*E. coli* (Type strain)	2.0 ± 6.2	19 (S)	10 (NA)	18 (NA)	15.7 ± 4.9	.0000	.0013	.0000	.0000

Key: Amk—Amikacin, Van—Vancomycin, Cli—Clindamycin, and NA—Not applicable. Interpretive categories: S—Sensitive (Amikacin ≥21 mm and Clindamycin ≥30 mm), R—Resistant (Amikacin ≤12 mm), and I—Intermediate (Clindamycin = 19 mm).

For the type strain *S. aureus* isolate, the mean zone of inhibition of the crude extract (15.6 ± 5.8 mm) was not significantly different (*p* = .4064) from the vancomycin reference zone (16 mm) (Table 2). It was also not significantly different (*p* = .0625) from the mean reference zone (18.7 ± 2.5 mm).

For the type strain *P. aeruginosa* strain, the mean zone of inhibition of the crude extract (22.6 ± 4.4 mm) was not significantly different (*p* = .8606) from the amikacin reference zone (20 mm) (Table 2). It was also not significantly different (*p* = .9855) from each of the vancomycin reference zone (18 mm) and the mean reference zone (22.0 ± 5.3 mm).

The clinical *E. coli* was completely resistant to the crude propolis extract, unlike the commercial reference antibiotic disk (Table 2).

### Antimicrobial susceptibility testing of the propolis fractions

The CH _AR_ fraction showed antimicrobial activity against the type strain *S. aureus* (8 mm), but did not show any antimicrobial activity against the clinical *S. aureus* (0 mm) isolate (Table 3). The CH _VR_ fraction did not show any antimicrobial activity against the clinical *S. aureus* isolate (0 mm), but did against the type strain *S. aureus* (12 mm). The EA _ER_ fraction did not show any antimicrobial activity against the clinical *S. aureus* isolate (0 mm), but did against the type strain *S. aureus* isolate (11 mm).

**Table 3.** Zones of inhibition produced by the propolis fractions.

Propolis	Zone diameter (mm)					
Fraction	*S. aureus* (clinical)	*S. aureus* (type strain)	*P. aeruginosa* (clinical)	*P. aeruginosa* (type strain)	*E. coli* (clinical)	*E. coli* (type strain)
CH_NR_	8.0 ± 0.0	9.0 ± 0.0	23.5 ± 0.7	26.5 ± 0.7	8.5 ± 0.7	8.0 ± 0.0
CH_ER_	0.0 ± 0.0	9.0 ± 0.0	25.0 ± 0.0	25.0 ± 0.0	0.0 ± 0.0	8.5 ± 0.7
CH_AR_	0.0 ± 0.0	8.0 ± 0.0	9.0 ± 0.0	12.0 ± 0.0	0.0 ± 0.0	12.0 ± 0.0
CH_VR_	0.0 ± 0.0	12.0 ± 0.0	12.0 ± 0.0	12.0 ± 0.0	0.0 ± 0.0	13.5 ± 0.7
EA_NR_	9.0 ± 0.0	11.5 ± 0.7	0.0 ± 0.0	9.0 ± 0.0	0.0 ± 0.0	0.0 ± 0.0
EA_ER_	0.0 ± 0.0	11.0 ± 0.0	0.0 ± 0.0	32.0 ± 0.0	16.0 ± 0.0	16.0 ± 0.0
EA_AR_	10.0 ± 0.0	27.0 ± 0.0	0.0 ± 0.0	31.0 ± 1.4	8.0 ± 0.0	10.0 ± 0.0
EA_VR_	27.0 ± 0.0	27.0 ± 0.0	12.5 ± 0.7	30.5 ± 0.7	18.0 ± 2.8	18.0 ± 0.0
PET_NR_	0.0 ± 0.0	0.0 ± 0.0	0.0 ± 0.0	0.0 ± 0.0	0.0 ± 0.0	0.0 ± 0.0
PET_ER_	0.0 ± 0.0	0.0 ± 0.0	0.0 ± 0.0	0.0 ± 0.0	0.0 ± 0.0	0.0 ± 0.0
PET_AR_	0.0 ± 0.0	0.0 ± 0.0	0.0 ± 0.0	0.0 ± 0.0	0.0 ± 0.0	0.0 ± 0.0
PET_VR_	9.0 ± 0.0	9.5 ± 0.7	0.0 ± 0.0	0.0 ± 0.0	0.0 ± 0.0	9.0 ± 0.0

Key: CH_NR_—Chloroform fraction of the Northern Region; CH_ER_—Chloroform fraction of the Eastern Region; CH_AR_—Chloroform fraction of the Ashanti Region; CH_VR_—Chloroform fraction of the Volta Region; EA_NR_—Ethyl acetate fraction of the Northern Region; EA_ER_—Ethyl acetate fraction of the Eastern Region; EA_AR_—Ethyl acetate fraction of the Ashanti Region; EA_VR_—Ethyl acetate fraction of the Volta Region; PET_NR_—Petroleum ether fraction of the Northern Region; PET_ER_—Petroleum ether fraction of the Eastern Region; PET_AR_—Petroleum ether fraction of the Ashanti Region, and PET_VR_—Petroleum ether fraction of the Volta Region.

The EA_NR_ did not show any antimicrobial activity against the clinical *P. aeruginosa* (0 mm) isolate, but did against the type strain *P. aeruginosa* (9 mm) (Table 3). Similarly, the EA_ER_ did not show any antimicrobial activity against the clinical *P. aeruginosa* isolate (0 mm), but did against the type strain *P. aeruginosa* (32 mm). Likewise, the EA_AR_ did not show any antimicrobial activity against the clinical *P. aeruginosa* (0 mm), but did against the type strain *P. aeruginosa* (32 mm). However, the PET_VT_ did not show antimicrobial activity against the clinical *P. aeruginosa* and the type strain *P. aeruginosa*.

The CH_ER_ showed no difference in antimicrobial activity of the fraction against the type strain *E. coli* and the clinical *E. coli* isolate (0 mm) (Table 3). The CH_AR_, however, did not have any antimicrobial activity against the clinical *E. coli* (0 mm) but had activity against the type strain *E. coli* (12 mm). Similarly, the CH_VR_ did not have any antimicrobial activity against the clinical *E. coli* isolate (0 mm), but did against type strain *E. coli* (14 mm). In contrast, the EA_NR_ did not have any antimicrobial activity against both the clinical and the type strain *E. coli* isolates (0 mm). The EA_AR_ fraction also did not show any antimicrobial activity against both the clinical and the type strain *E. coli* isolates (0 mm). The PET_VT_ had antimicrobial activity against the type strain *E. type strain* (9 mm), but did not have antimicrobial activity against the clinical *E. coli* isolates.

The PET_NR_, PET_ER_, and PET_AR_ fractions did not show antimicrobial activity against any of the test isolates (0 mm).

### MIC of the effective propolis fractions

The effective propolis fractions were the CH_NR_, CH_ER_, EA_ER_, EA_VR_, and EA_AR_ (Table 4; Fig. 2B). There was no significant difference (*p* = .8554) in the mean MIC of the effective propolis fractions against the clinical *S. aureus* (76.0 ± 34.8 mg/ml) and the type strain *S. aureus* (48.8 ± 33.0 mg/ml). Similarly, there was no significant difference (0.7168) in the mean MIC of the effective propolis fractions against the clinical *P. aeruginosa* (40.8 ± 33.2 mg/ml) and the type strain *P. aeruginosa* (30.4 ± 6.7 mg/ml). The MIC of the effective propolis fractions against the clinical and the type strain *E. coli* were, however, not within the range of the tested dilutions, and as such, the means were not reported.

**Table 4.** MIC (mg/ml) of the effective propolis fractions.

Effective	MIC (mg/ml)					
Propolis	*S. aureus*		*P. aeruginosa*		*E. coli*	
Fraction	Clinical	Type strain	Clinical	Type strain	Clinical	Type strain
CH_NR_	56	100	24	40	64	–
CH_ER_	100	64	32	32	–	–
EA_ER_	100	24	24	32	–	–
EA_AR_	100	32	100	24	–	40
EA_VR_	24	24	24	24	32	–
Mean ± SD	76.0 ± 34.8	48.8 ± 33.0	40.8 ± 33.3	30.4 ± 6.7	NA	NA
5% DMSO	–	–	–	–	–	64

Key: – —No activity within the range of the tested dilutions, NA—Not applicable. CH_NR_—Chloroform fraction of the Northern Region; CH_ER_—Chloroform fraction of the Eastern Region; EA_ER_—Ethyl acetate fraction of the Eastern Region; EA_AR_—Ethyl acetate fraction of the Ashanti Region; and EA_VR_—Ethyl acetate fraction of the Volta Region.

### MBC of the effective propolis fractions

For the effective propolis fractions CH_NR_, CH_ER_, EA_ER_, EA_VR_, and EA_AR_ (Table 5; Fig. 2C), there was no significant difference (*p* = .8913) between the mean MBC against the clinical *S. aureus* (79.2 ± 30.7 mg/ml) and the type strain *S. aureus* (50.4 ± 31.7 mg/ml). Similarly, there was no significant difference (*p* = .7256) between the mean MBC against the clinical *P. aeruginosa* (45.6 ± 31.5 mg/ml) and the type strain *P. aeruginosa* (28.0 ± 43.8 mg/ml). The MBCs of the effective propolis fractions against the clinical and the type strain *E. coli* were not within the range of the tested dilutions, and as such, the means were not reported.

**Table 5.** MBC (mg/ml) of the effective propolis fractions.

Effective	MBC mg/ml					
Propolis	*S. aureus*		*P. aeruginosa*		*E. coli*	
Fraction	Clinical	Type strain	Clinical	Type strain	Clinical	Type strain
CH_NR_	64	100	40	40	100	–
CH_ER_	100	64	40	32	–	–
EA_ER_	100	32	24	32	–	–
EA_AR_	100	32	100	24	–	40
EA_VR_	32	24	24	24	40	–
Mean ± SD	79.2 ± 30.7	50.4 ± 31.7	45.6 ± 31.5	28.0 ± 43.8	NA	NA
5% DMSO	–	–	–	–	–	100

Key:– —No activity within the range of the tested dilutions. NA—Not applicable; CH_NR_—Chloroform fraction of the Northern Region; CH_ER_—Chloroform fraction of the Eastern Region; EA_ER_—Ethyl acetate fraction of the Eastern Region; EA_AR_—Ethyl acetate fraction of the Ashanti Region; and EA_VR_—Ethyl acetate fraction of the Volta Region.

## Discussion

The crude propolis extracts and fractions from the various regions showed different degrees and patterns of antimicrobial activity against the tested bacterial isolates. This is because the antimicrobial activities of propolis vary by geographical origin and the vegetation available for the bees producing the propolis (Kumar et al. 2008). Different plants contain different phytochemical constituents and this in turn influences the composition of the final propolis products produced by the bees. This may account for the different degrees and patterns of activity shown by both the crude propolis extracts and their corresponding solvent fractions. Therefore, the crude propolis extract from Volta Region had the most active phytochemical composition responsible for its relatively greater antimicrobial activity against the test isolates.

Plants contain polyphenols which are known for their antimicrobial properties. Therefore, propolis also contain polyphenols (Przybylek and Karpinski 2019) since these are derived from plants. The antimicrobial activity of plant polyphenols depends on the type and concentration of each polyphenol present in the extract (Manso et al. 2022). These have been shown to produce synergistic antimicrobial activity in combination with conventional antimicrobial drugs (Manso et al. 2022). The actual polyphenols present in various propolis sources should be elucidated to identify the most significant ones, which may have industrial applications in terms of producing new antimicrobial drugs. Notable among these are the flavonoids, which target various bacterial cell components as well as nucleic acid synthesis and may account for much of the known antimicrobial activity (Almuhayawi 2020). Apart from the flavonoids, propolis also contains terpenoids (Huang et al. 2014), which may also contribute to its antimicrobial potential.

Despite the chemical heterogeneity of propolis from different sources (Peixoto et al. 2022), it is currently possible to standardize propolis extracts, and this has been successfully applied in some clinical trials (Salatino 2022). Since propolis is used in the food industry (Pobiega et al. 2019) polyphenols from propolis can be incorporated into oral drugs as an alternative treatment or used in combination therapy. Polyphenols are poorly digested, so can achieve high concentrations in the gut (Dryden et al. 2006). Therefore, it should be possible to use propolis-based drugs for treatment of antimicrobial-resistant gastroenteric infections. Apart from systemic infections, propolis may also have application in the production of topical drugs despite its low solubility, which may make it unappealing to the pharmaceutical industry. An et al. (2021) have shown that the poor solubility can be remarkably improved by using poloxamers as solubilizers and gelling agents. Wagh (2013) has previously reported the use of propolis in the treatment of upper respiratory, wound, and skin infections, which account for the use of many conventional antimicrobial drugs. The addition of propolis-based drugs to the battery of conventional antimicrobial drugs available may reduce over-dependence on these drugs and subsequently reduce the rate of the increasing antimicrobial-resistance menace.

The antimicrobial activity of crude propolis extract may have potential therapeutic use for the treatment *P. aeruginosa* and *S. aureus*-associated infections. The MBCs of the effective fractions against the test isolates of *S. aureus* and *P. aeruginosa* suggests that the CH and EA fractions may have a broad bactericidal effect against different strains of these species of bacteria. Although ethanol was not used for extraction in the present study, the MIC of 24–100 mg/ml obtained for the EA and CH solvent extracts of propolis tested against Gram-positive bacteria in this study was much greater than the 0.08–2.5 mg/ml range reported by Al-Ani et al. (2018) for propolis sampled from Europe. It is evident that the MIC and the MBC of Egyptian, Brazilian, and Serbian propolis were lower than the MIC and MBC of Ghanaian propolis. Studies have revealed varying antimicrobial activity of propolis fractions from different sources (Kumar et al. 2008). El-Fadaly and El-Badrawy (2001) disclosed that the MIC and the MBC of EA fraction of the Egyptian propolis against *S. aureus* and *E. coli* were 0.75 mg/ml and 0.85 mg/ml, respectively. However, CLSI (2016) has reported that the tube dilution method gives more reproducible results in the determination of the antimicrobial activity of natural products with poor water solubility than the agar well diffusion method. Apart from the difference in nature of the tested isolates, this may account for the differences observed.

This, however, may not be the case for *E. coli* for which higher concentrations were required in most cases for both the CH and EA fractions to produce any inhibitory effect. It has been reported that propolis from Europe has a moderate activity against Gram-negative bacteria with a reported MIC of 0.6–5 mg/ml for the ethanolic extract (Al-Ani et al. 2018). Propolis extract may interfere with cell wall composition or cell metabolic activities leading to the destruction of Gram-positive bacteria as observed in the case of *S. aureus*. Antibiotic resistance in *P. aeruginosa* is largely due to efflux pump inhibitors (Askoura et al. 2011). The antimicrobial extract may have overcome this mechanism of resistance leading to the destruction of *P. aeruginosa*. In the case of *E. coli*, a Gram-negative bacterium, the relatively higher resistance to the crude propolis extracts may be due to the lack of an effective target site in the metabolic or cellular structural components required for destruction. It has been reported that the antimicrobial activity of propolis extract is higher in Gram-positive bacteria than in Gram-negative bacteria (Przybylek and Karpinski 2019). Polyphenols may modify the permeability of cell membranes and destroy the rigidity and integrity of the bacterial cell wall (Bouarab-Chibane et al. 2019). The difference in the activity of propolis against Gram-positive and Gram-negative bacteria may be due to differences in the structure of the cell wall. Gram-negative bacteria have an outer membrane and a thinner peptidoglycan layer whereas Gram-positive bacteria do not have an outer membrane but possess a thicker peptidoglycan layer (Silhavy et al. 2010). The component affected may be the peptidoglycan. The outer membrane of the Gram-negative bacteria may limit the passage of the polyphenol component of propolis and prevent it from acting against the relatively thinner peptidoglycan layer in the Gram-negative bacteria. Therefore, crude propolis extracts may not have a strong therapeutic potential for treating *E. coli*-associated infections with a possible extension of resistance to the Enterobacteriaceae.

The potency of the different solvent extracts against the different bacteria differed. Nichitoi et al. (2021) reported that the polyphenolic profile of propolis is influenced by the solvent used for the extraction and this in turn influences the antimicrobial activity. Of the three solvents used, the best solvents for fractionating the antimicrobial constituents of the crude propolis extract were CH and EA. Since the petroleum ether fraction (PET) of the crude propolis from the different regions did not show antimicrobial activity in most cases this indicates that the fraction may have poor therapeutic potential for the treatment of infections caused by the test isolates. Silvana et al. (2009) have previously reported that the different chemical fractions of CH, EA, hexane, and ethanol investigated by several authors from North-Eastern Brazil have significant antimicrobial activities (*p* < .05). Therefore, increasing the working concentration of the crude propolis extract above 64 mg/ml may yield solvent fractions possessing relatively greater antimicrobial activity and hence improve the therapeutic value.

There is a strong potential for propolis to be used as an antimicrobial drug and this should be further explored as an alternative for treatment of bacterial infections. Although propolis varies between regions, qualitative testing can lead to the identification and isolation of the bioactive components which can be standardized and exploited for their antimicrobial potential. CH and EA fractions obtained from crude propolis extracts may serve as a potential source of therapeutic agents against *P. aeruginosa* and *S. aureus*-associated infections. Using these solvents for the extraction resulted in propolis fractions, which had greater antimicrobial activity than PE solvent extract. However, the limitation is that the extracts were tested only in biological duplicates and hence may require further replication. Also, Pobiega et al. (2019) reported that differences in the extraction method can influence the antimicrobial activity of the final product although this did not influence the qualitative composition of the final product. In this study, only one extraction method was applied although different solvents were used. It will be necessary to vary the extraction method to ascertain how this may impact on the antimicrobial activities of the final products. Also, Nichitoi et al. (2021) supports the idea of trying a 50% ethanolic extract but ethanolic extracts of propolis were not tested in this study to ascertain an additional antimicrobial potential of the sampled propolis. Further investigation is needed to isolate and elucidate the structure of the active compounds present in Ghanaian propolis to test for the possible synergy between the phytochemicals present and how these can be applied in the production of new antimicrobials Additionally, melliferous plants and trees around beehives should be screened for antimicrobial activities.

## Consent to participate

Not applicable.

## Data Availability

Not applicable.
